# Liver-targeted delivery of asiatic acid nanostructured lipid carrier for the treatment of liver fibrosis

**DOI:** 10.1080/10717544.2021.2008054

**Published:** 2021-12-02

**Authors:** Ya-Wen Zhang, Ling-Lan Tu, Yi Zhang, Jie-Chao Pan, Gao-Li Zheng, Li-Na Yin

**Affiliations:** a Institute of Materia Medica, Hangzhou Medical College, Hangzhou, China; b School of Biological Engineering, Hangzhou Medical College, Hangzhou, China; c Hangzhou Xianju Technology Innovation Co. Ltd, Hangzhou, China; d Safety Evaluation Research Center, Hangzhou Medical College, Hangzhou, China

**Keywords:** Liver-targeted therapy, nanostructured lipid carrier, asiatic acid, Box–Behnken design, liver fibrosis

## Abstract

Liver fibrosis is a major global health concern. Management of chronic liver disease is severely restricted in clinics due to ineffective treatment approaches. However, a lack of targeted therapy may aggravate this condition. Asiatic acid (AA), a pentacyclic triterpenoid acid, can effectively protect the liver from hepatic disorders. However, the pharmaceutical application of AA is limited by low oral bioavailability and poor targeting efficiency. This study synthesized a novel liver-targeting material from PEG-SA, chemically linked to ursodeoxycholic acid (UA), and utilized it to modify AA nanostructured lipid carriers (UP-AA-NLC) with enhanced targeting and improved efficacy. The formulation of UP-AA-NLC was optimized via the Box–Behnken Experimental Design (BBD) and characterized by size, zeta potential, TEM, DSC, and XRD. Furthermore, *in vitro* antifibrotic activity and proliferation of AA and NLCs were assessed in LX-2 cells. The addition of UP-AA-NLC significantly stimulated the TGF-beta1-induced expression of α-SMA, FN1, and Col I α1. *In vivo* near-infrared fluorescence imaging and distribution trials in rats demonstrated that UP-AA-NLC could significantly improve oral absorption and liver-targeting efficiency. Oral UP-AA-NLC greatly alleviated carbon tetrachloride-induced liver injury and fibrosis in rats in a dosage-dependent manner, as reflected by serum biochemical parameters (AST, ALT, and ALB), histopathological features (H&E and Masson staining), and antioxidant activity parameters (SOD and MDA). Also, treatment with UP-AA-NLC lowered liver hydroxyproline levels, demonstrating a reduction of collagen accumulation in the fibrotic liver. Collectively, optimized UP-AA-NLC has potential application prospects in liver-targeted therapy and holds great promise as a drug delivery system for treating liver diseases.

## 1. Introduction

Liver fibrosis is characterized by excessive deposition of extracellular matrix (ECM) and dysfunction of sinusoidal endothelial cells following chronic liver damage (Shouval & Friedman, 2014). This condition remains a global health problem. If not timely treated, the liver parenchyma is replaced by scar tissue, causing cirrhosis or liver cancer. At present, no anti-fibrosis drugs have been approved. The frequently used drugs in clinics are ineffective due to a lack of organ selectivity and the inability to deliver sufficient drug concentration to the lesion. Additionally, a lack of liver-targeting therapies causes toxicity and side effects. Therefore, it is imperative to develop new drug delivery systems with effective targeting abilities, high accuracy, and low toxicity.

Nanotechnology, which enables passive targeted delivery of drugs to the liver, has attracted increasing attention in recent years (Mishra et al., 2010; Doane & Burda, 2013). Nanostructured lipid carrier (NLC) is a promising nanotechnology-based drug delivery system that potentially improves the *in vivo* pharmacokinetics and bioavailability of water-insoluble drugs. Compared to traditional lipid carrier delivery systems (such as SLN), the NLC demonstrates better encapsulation and stability as it comprises a mixture of solid–liquid lipids with varying melting points (Müller et al., 2002; Shidhaye et al., 2008). However, the application of NLC in the treatment of liver diseases is limited by low hydrophilicity and lack of active targeting.

Bile acids are endogenous hepatocyte-specific natural ligands, actively transported to hepatocytes via Na^+^/taurocholate cotransporter (NTCP) on the surface of hepatocytes (Sievanen, 2007). Bile acids can serve as small molecule liver-targeting carriers for oral absorption. Furthermore, bile acids contribute to human hepatoenteric circulation and demonstrate a powerful transport capability (Dawson, 2011). Compelling evidence shows that combining bile acids with insoluble drugs can promote liver aggregation and improve oral bioavailability by increasing intestinal membrane permeability and lymphatic transport (Chen et al., 2009, 2010; Zhang et al., 2013; Xiao et al., 2019). Ursodeoxycholic acid (UA), one of the main components of bile acids, is biocompatible and is absorbed from the intestine into the liver via the active transport pathway after oral administration. Of note, UA has a high transport capacity and organ-specific absorption characteristics, which potentially increase the drug concentration in the liver, prolong circulating time, and reduce side effects (Fiorucci et al., 2001, 2004; Paschke et al., 2003). Studies have also demonstrated the pharmacological activities of UA in protecting the liver (Ye et al., 2020). Therefore, UA can be utilized as a drug carrier and may exert a synergistic role to improve the treatment efficiency of liver diseases.

Traditional Chinese Medicine has excellent advantages in anti-liver fibrosis with its unique view of syndrome differentiation and holistic treatment theory (Zhang & Schuppan, 2014). Asiatic acid (AA) is the main component of *Centella asiatica*. Evidence indicates that AA can reverse liver fibrosis, relieve liver damage, and has significant hepatoprotective effects (Gao et al., 2006; Pakdeechote et al., 2014). The mechanism of AA in liver fibrosis inhibition is associated with anti-mitochondrial stress, cellular antioxidant, and blocking TGF-β1 autocrine (Mi-Sook et al., 2004; Tang et al., 2012). However, due to the extremely low solubility in water (≈10 μg/mL) and rapid elimination *in vivo*, the oral bioavailability of AA is extremely low (Chasseaud et al., 1971; Rush et al., 1993). Furthermore, research evidence shows that AA is widely distributed in tissues and organs after administration, showing dose and time dependence, severely limiting its clinical application. In this view, it would be imperative to develop effective liver-targeted drug delivery systems to improve the bioavailability and therapeutic effect of AA. Our previous investigation developed a PEG-modified AA NLC (P-AA-NLC), which passively targeted the liver and improved oral absorption (Chen et al., 2020). Herein, we modified an AA NLC with UA to evaluate the therapeutic effects of UA-modified UP-AA-NLC on liver targeting and anti-fibrosis activity in carbon tetrachloride (CCl_4_)-induced liver fibrosis rat model.

## 2. Materials and methods

### 2.1. Chemicals and animals

Asiatic acid (98%) was purchased from Guangxi Institute for Food and Drug Control (Guangxi, China). Glycyrrhetinic acid (IS, >99%) was acquired from Shanghai Chenyi Biological Technology Co., Ltd. (Shanghai, China). β-Glucuronidase (Type B-1, ≥1,000,000 units/g) was procured from Sigma-Aldrich (St. Louis, MO). PEG_2000_-SA was purchased from Kasei Kogyo Co., Ltd. (Tokyo, Japan). Ursodeoxycholic acid (99%) was purchased from Aladdin (Shanghai, China). Near-infrared fluorescent dye DIR was acquired from Dalian Meilun Biotechnology Co., Ltd. (Dalian, China). Colchicine was procured from Guangdong Peter Medicine Co., Ltd. (Guangdong, China). Carbon tetrachloride was purchased from Sinopharm Chemical Reagent Co., Ltd. (Shanghai, China). The aspartate aminotransferase (AST), albumin (ALB), and glutamic-pyruvic transaminase (ALT) detection assay kits were procured from Fuxing Changzheng Medical Science Co., Ltd. (Shanghai, China). The superoxide dismutase (SOD), malondialdehyde (MDA), and hydroxyproline (HYP) detection assay kits were purchased from Nanjing Jiancheng Biotechnology Institute (Nanjing, China). All chemicals and reagents were of analytical or HPLC grade.

Male Sprague-Dawley (SD) rats (200 ± 20 g) and ICR mice (4–5 weeks) were purchased from Zhejiang Laboratory Animal Center (Hangzhou, China) and maintained at 22 ± 2 °C in a 12-h light/12-h dark cycle. The animal experiments adhered to laboratory animal care principles and were approved by the Institutional Animal Ethical Committee of Zhejiang (China).

### 2.2. Preparation and characterization of UA-PEG-SA

**Figure 1.**
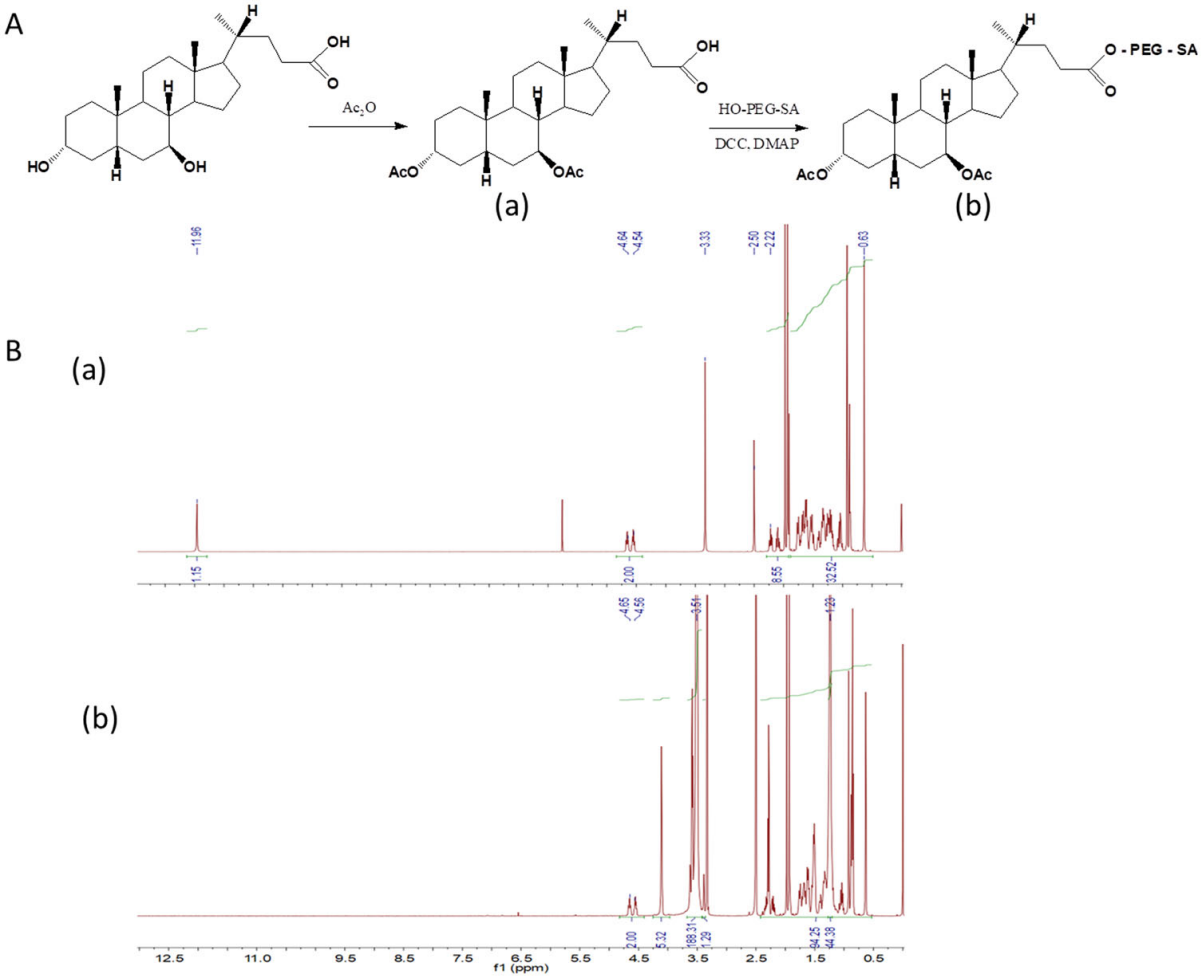
Synthesis scheme (A) and ^1^H-NMR spectra (B) of UA-PEG-SA conjugate. (a) Acetylated ursodeoxycholic acid; (b) UA-PEG-SA conjugate.

The chemical reaction scheme for UA-PEG-SA conjugate preparation is illustrated in Figure 1(A). Briefly, the hydroxyl group on UA was reacted with acetic anhydride to form an ester for protection. The resultant acetylated UA was reacted with PEG-SA at room temperature to form esters, catalyzed by DCC and DMAP.

#### 2.2.1. Synthesis of acetylated ursodeoxycholic acid

Ursodeoxycholic acid (15.7 g, 40 mmol), acetic acid (4 mL), and acetic anhydride (120 mL) were added into a round bottom flask, and refluxed at 140 °C by a magnetic stirrer. Samples were collected at varying times, diluted 10 times with dichloromethane, and monitored via TLC (CH_2_Cl_2_:CH_3_OH:CH_3_COOH = 50:1:0.05). Eight hours later, the solvent was removed by evaporation on a rotary evaporator (Rotavapor R-210, Buchi, Flawil, Switzerland). The crude product was dissolved in dichloromethane, mixed with an equal volume of water, and stirred for 4 h. The dichloromethane layer was removed. Subsequently, an equal amount of water was added and stirred for 4 h. These steps were repeated thrice. Finally, an appropriate amount of anhydrous sodium sulfate was added to the dichloromethane layer, stirred overnight, and filtered. The filtrate was evaporated to dryness on a rotary evaporator. The resultant oily substance was purified on silica gel column (CH_2_Cl_2_:CH_3_OH = 100:1, v/v) and the TLC *R*_f_=0.28 (CH_2_Cl_2_:CH_3_OH:CH_3_COOH = 50:1:0.05, color developed with sulfuric acid) was collected.

#### 2.2.2. Synthesis of UA-PEG-SA

Acetylated UA 1.7 g (3.75 mmol) and PEG-SA 7.5 g (3.75 mmol) were weighed into a round bottom flask. Next, 150 mL of toluene was added, and the mixture refluxed until no water droplets appeared in the water separator (125 °C for 5 h, magnetic stirred). The mixture was dried under reduced pressure. After that, DCC (1.5 g), DMAP (0.1 g), and dichloromethane were added and stirred for two days at 25 °C. The filtrate was washed thrice with saturated NaHCO_3_ and NaCl. An appropriate amount of anhydrous sodium sulfate was added to the dichloromethane layer and soaked overnight. The filtrate was decompressed to remove the solvent and purified further on a silica gel column (CH_2_Cl_2_:CH_3_OH = 25:1, v/v). The single point *R*_f_=0.68 (CH_2_Cl_2_:CH_3_OH = 10:1) analyzed by TLC was visualized with sulfuric acid. Finally, UA-PEG-SA was vacuum dried at room temperature and recovered as a waxy solid. The acquired solid was dissolved in DMSO-d_6_ and analyzed by ^1^H NMR (AC-80, Bruker Biospin Co., Ettlingen, Germany).

### 2.3. Preparation and characterization of AA-loaded nanostructured lipid carriers

#### 2.3.1. Preparation of P-AA-NLC and up-AA-NLC

The AA-loaded NLC modified with PEG-SA (P-AA-NLC) and UP-PEG-SA (UP-AA-NLC) was prepared using solvent diffusion method (Chen et al., 2020). Briefly, AA, glyceryl monostearate, oleic acid, PEG-SA, or UP-PEG-SA were accurately weighed and dissolved in ethanol (70 °C). Subsequently, the mixture was added into distilled water at the same temperature under a mechanical stirrer at 500 rpm. The organic solvent in the dispersion was removed under reduced pressure at 40 °C. The prepared NLC was centrifuged (at 5000 rpm) for 10 min to remove unwrapped AA (AnkeGL-20G-II, Shanghai, China). Fluorescent NLCs were prepared by DIR and utilized in the *in vivo* distribution studies.

#### 2.3.2. Experimental design

A Box–Behnken design (BBD) with three factors and three levels was utilized for UP-AA-NLC optimization. Fitting analysis on the test data was achieved with Design Expert 8.0.6 (Stat-Ease, Inc., Minneapolis, MN). According to preliminary results, three independent factors significantly influenced NLC formulation, including AA/lipid (*A*, 5–30%), UA-PEG-SA/lipid (*B*, 5–20%), and oleic acid/lipid (*C*, 10–30%). The four dependent variables included particle size (*Y*_1_), zeta potential (*Y*_2_), encapsulation efficiency (EE) (*Y*_3_), and drug loadings (DLs) (*Y*_4_). The factor levels were coded as (−1, 0, and 1) as listed in Table 1. The experimental design and response value results of Box–Behnken are displayed in Table 2. Analysis of variance (ANOVA) was applied to evaluate the significance of each factor by *p* value (<.05) and select the appropriate model. The polynomial equation generated for this experimental design is as follows:

(1) $$Y\;=b_{0}+\;b_{1}A\;+\;b_{2}B\;+\;b_{3}C\;+\;b_{12}AB\;+\;b_{13}AC\;+\;b_{23}BC+\;b_{11}A^{2}+\;b_{22}B^{2}+\;b_{33}C^{2}$$

where *Y* is the dependent variable; *b*_0_ is the intercept; *b*_1_ to *b*_33_ are the regression coefficients; *A*, *B*, and *C* are the factors being investigated.

**Table 1.** Factors and levels.

Factors	Levels		
	Low (–1)	Medium (0)	High (1)
*A* (AA/lipid, %w/w)	5.0	17.5	30.0
*B* (UA-PEG-SA/lipid, % w/w)	5.0	12.5	20.0
*C* (oleic acid/lipid, % w/w)	10.0	20.0	30.0

**Table 2.** Design and response values of the Box–Behnken test (*n* = 3).

Run	Std	*A*, %	*B*, %	*C*, %	*Y*_1_, nm	*Y*_2_, mV	*Y*_3_, %	*Y*_4_, %
1	12	0	1	1	192.3	–19.2	86.72	11.11
2	5	–1	0	–1	167.1	–22.9	90.30	3.65
3	3	–1	1	0	173.9	–28.0	84.55	3.53
4	14	0	0	0	183.2	–24.9	83.63	10.94
5	2	1	–1	0	158.8	–21.7	70.50	15.50
6	7	–1	0	1	171.5	–26.6	77.57	3.37
7	15	0	0	0	183.2	–24.9	83.63	10.94
8	13	0	0	0	183.2	–24.9	83.63	10.94
9	17	0	0	0	183.2	–24.9	83.63	10.94
10	6	1	0	–1	179.9	–19.9	77.81	16.25
11	1	–1	–1	0	160.9	–31.2	85.11	4.20
12	4	1	1	0	195.0	–27.0	69.65	13.94
13	10	0	1	–1	178.0	–18.7	83.05	10.42
14	9	0	–1	–1	150.9	–21.6	80.80	11.62
15	8	1	0	1	165.9	–26.1	77.13	15.54
16	16	0	0	0	183.2	–24.9	83.63	10.94
17	11	0	–1	1	158.2	–27.9	79.90	11.45

*Y*_1_: particle size: nm; *Y*_2_: zeta potential, mV; *Y*_3_: encapsulation efficiency, %; *Y*_4_: drug loadings, %.

#### 2.3.3. Characterization of up-AA-NLC

The mean particle size, zeta potential, and polydispersity index (PI) of NLCs were detected by Zetasizer Nano ZS90 (Malvern, Malvern, UK) at a constant temperature of 25 °C. Freshly prepared samples were dispersed in ultrafiltration water and ultrasonically dispersed for 3 min (JY92-2D, Scientz Biotechnology Co. Ltd., Ningbo, China).

The NLC suspension was centrifuged to remove free AA because of the low water solubility of AA. The supernatant (1 mL) was precisely pipetted, diluted to 25 mL with ethanol, and heated for 30 min at 70 °C. Filtered samples were analyzed via HPLC as described previously (Chen et al., 2020). The HPLC system comprised a binary LC-20AD pump (Shimadzu, Kyoto, Japan), an SPD-20A UV-vis detector (Shimadzu, Kyoto, Japan), and Diamonsil^TM^ C_18_ column (5 μm, 250 mm × 4.6 mm). The mobile phase was prepared with methanol/acetonitrile (1:1, v/v) and 0.02 mol/L KH_2_PO_4_ (pH 3.0) at a volume ratio of 80/20. The detection wavelength and the flow rate were set at 210 nm and 1.0 mL/min, respectively. The column temperature was thermostated at 25 °C. The EE (%) and DLs (%) were calculated according to formulas (2) and (3):

(2) $$\mathrm{EE (\%)}=\frac{\mathrm{amount of drug in NLC}}{\mathrm{total drug added}}\times \mathrm{100\%}$$

(3) $$\mathrm{DL (\%)}=\frac{\mathrm{amount of drug in NLC}}{\mathrm{weight of NLC}}\times \mathrm{100\%}$$

The morphology of NPs was examined using a transmission electron microscope (TEM; JEM-1200EX, JEOL, Tokyo, Japan). Briefly, the sample was negatively stained with 2% phosphotungstic acid (w/v) and placed on a copper mesh for observation.

DSC was performed by Mettler DSC1 differential scanning calorimeter (Mettler Toledo, Greifensee, Switzerland). The samples (AA powder, ingredients mixtures, physical mixtures, UP-AA-NLC, and P-AA-NLC) were weighed accurately and heated to 300 °C at a heating rate of 10 °C/min to record DSC scans.

The crystal form of the formulations was evaluated via powder X-ray diffraction (XRD) with a powder X-ray diffractometer (D8ADVANC, Bruker, Karlsruhe, Germany). XRD patterns of AA powder, ingredients mixtures, physical mixtures, UP-AA-NLC, and P-AA-NLC, were recorded. Samples were scanned at a scanning rate of 0.02°/s in the 2*θ* range from 3° to 40°.

The physical stability of UP-AA-NLC was examined by monitoring the size changes at different time points under 4 °C storage conditions. The stability of UP-AA-NLC in serum was also investigated. Freshly prepared UP-AA-NLC was dispersed in 10 mL pH 7.4 PBS (containing 10% fetal bovine serum) and incubated at 37 °C. Samples were collected at a predetermined time to measure the particle sizes.

### 2.4. *In vitro* release of AA

*In vitro* release of AA, P-AA-NLC, and UP-AA-NLC was conducted in pH 7.4 PBS (1% SDS, w/v) by the dialysis bag technology (MWCO: 3500 Da, Wuhan Xinsirui Technology Co., Ltd., Wuhan, China). The temperature of the oscillator (HZ-9211KB, HuaLiDa Laboratory Equipment Company, Jiangsu, China) was set to 37 °C at stirring rate of 100 rpm. An aliquot (1 mL) was collected at scheduled time points and replaced with an equal volume of PBS (pH 7.4). All samples were filtered and analyzed by HPLC as described previously.

To explore the AA release mechanism of NLCs, the *in vitro* release data were fitted to the Ritger–Peppas model (Ritger & Peppas, 1987), which has high fitness for the nanoparticle release process (Cunha et al., 2020; Wu et al., 2021):

(4) $$\frac{M_{t}}{M_{\infty }}=Kt^{n}$$

where *M_t_* and *M*_∞_ denoted the cumulative AA release at time t and infinite time, respectively; the release mechanism was reflected by release exponent *n*; *k* is the release constant. If *n* = 0.45, the release mechanism corresponded to Fickian’s diffusion; when 0.45<*n* < 0.89, non-Fickian (anomalous) drug diffusion occurs; *n* = 0.89 represents case II transport, and *n* > 0.89 represents super case II transport (Jamwal et al., 2019).

### 2.5. Cell proliferation experiment

The proliferation of LX-2 cells was evaluated by a cell counting kit-8 (CCK-8; Beyotime, Haimen, China). Briefly, LX-2 cells were seeded into 96-well plates (6 × 10^4^ cells/well), and incubated for 12 h. The cells were subsequently incubated with different concentrations of AA or UP-AA-NLC for 24 h (0, 4, 10, and 25 μM). The OD_450_ values of each well were recorded using a SpectraMax iD5 microplate reader (Molecular Devices, Sunnyvale, CA). Wells untreated with drugs represented 100% cell viability.

### 2.6. Quantitative real-time polymerase chain reaction (q-PCR) experiment

LX-2 cells were seeded into six-well plates (2 × 10^5^ cells/well) and pretreated with TGF-beta1 (R&D System, Minneapolis, MN) overnight at 10 ng/mL concentration. This was followed by the addition of different concentrations of AA or UP-AA-NLC for 48 h (0, 4, 10, and 25 μM). Total RNA was extracted using TRIzol reagent (Invitrogen, Carlsbad, CA). RNA concentration was assessed by NanoDrop2000 spectrophotometer (Thermo Scientific, Waltham, MA). The mRNA expression of Actin, α-smooth muscle actin (α-SMA), fibronectin 1 (FN1), and collagen type I alpha 1 chain (Col I α1) was quantified via SYBR-based quantitative PCR analysis (Hieff^®^ qPCR SYBR^®^ Green Master Mix, Yeasen Biotech Co., Ltd., Shanghai, China) on a programmed My Cycler (BIO-RAD, Feldkirchen, Germany). Thermocycling conditions were as follows: 95 °C for 10 min, followed by 40 cycles of 95 °C for 15 s and 60 °C for 1 min. Melting curve conditions were 95 °C 40 s, and the temperature was dropped to 60 °C in 1 min. The sequences of primers used were as follows: actin (human) forward: 5′-CTCCATCCTGGCCTCGCTGT-3′, reverse: 5′-GCTGTCACCTTCACCGTTCC-3′; α-SMA (human) forward: 5′-TTCAATGTCCCAGCCATGTA-3′, reverse: 5′-GCAAGGCATAGCCCTCATAG-3′; FN1 (human) forward: 5′-ATCACCCTCACCAACCTCAC-3′, reverse: 5′-TCCCTCGGAACATCAGAAAC-3′; Col I α1 (human) forward: 5′-ACTGGTGAGACCTGCGTGTA-3′, reverse: 5′-GAATCCATCGGTCATGCTCT-3′. The data were analyzed using the comparative 2^–ΔΔCt^ method. The PCR test was performed in triplicate.

### 2.7. *In vivo* near-infrared fluorescence imaging

Free DIR, DIR-labeled P-AA-NLC, and UP-AA-NLC were used to study the *in vivo* targeting efficacy after modification. Briefly, 15 ICR mice were divided randomly into three groups, and orally administered with different formulations at a 20 mg DIR/kg dose. The mice were anesthetized at predefined time points. The fluorescence of the formulations was visualized by a living imaging system (Clairvivo OPT, Shimadzu, Kyoto, Japan) at excitation and emission wavelengths of 748 nm and 780 nm, respectively. The exposure time was 30 seconds per cube. Finally, mice were sacrificed, and their hearts, livers, spleens, lungs, and kidneys were resected. NIR fluorescence signal intensity of different tissues was measured to examine the biological distribution of NLCs.

### 2.8. Tissue distribution in rats

A highly sensitive HPLC method was developed to detect the concentration of drugs in the major organs after oral administration of AA or UP-AA-NLC. The HPLC system contained a binary pumps, UV-vis detector (Shimadzu, Kyoto, Japan), and a C_8_ column (5 μm, 150 mm × 4.6 mm). The HPLC mobile phase solvent A contained distilled water, and solvent B was methanol–acetonitrile (1:1). The gradient elution procedure was as follows: 0–10 min, 25% A, 75% B; 10–25 min, 11% A, 89% B → 25% A, 75% B; 25–27 min, 25% A, 75% B. The UV wavelength, the flow rate, and column temperature were set at 248 nm, 1.0 mL/min, and 25 °C, respectively.

*In vivo*, near-infrared fluorescence imaging results showed that UP-AA-NLC reached peak concentration at 24 h. In this view, the concentration of AA, P-AA-NLC, and UP-AA-NLC, at 64 mg/kg dosage, was determined in ICR mice at 24 h. The heart, liver, spleen, lung, and kidney were excised and homogenized in normal saline. Subsequently, β-glucuronidase was added and hydrolyzed at 37 °C for 24 h. Glycyrrhetinic acid served as the internal standard. The mixture was extracted with methyl tert-butyl ether and centrifuged. The supernatant was collected, dried with nitrogen, and derivatized following a previously described protocol (Zheng & Wang, 2009). The concentration of AA in each organ was quantified under the above HPLC conditions.

### 2.9. Animal model of CCl_4_-induced liver fibrosis and experimental design

Seventy SD rats who underwent adaptive feeding for a week were divided randomly into seven groups (*n* = 10 for each group) including: (1) normal group, (2) liver fibrosis group, (3) colchicine group (0.1 mg/kg), (4) free AA group (32 mg/kg), and (5–7) UP-AA-NLC groups (16 mg/kg, 32 mg/kg, and 64 mg/kg, respectively). Except for the normal group, rats were intraperitoneally injected with 20% CCl_4_ oil solution (2 mL/kg, twice a week) for 6 weeks to induce liver fibrosis. Rats in groups 3 and 4 were orally administered with colchicine (0.1 mg/kg) or AA (32 mg/kg) daily for six consecutive weeks. Rats in groups 5, 6, and 7 were administered orally with different concentrations of UP-AA-NLC. The body weight was monitored weekly for all rats. At the end of the experiment, the rats were sacrificed, and blood samples were collected to analyze the liver function and fibrosis indicators. Liver specimens from all groups were weighed and fixed with 10% formaldehyde for subsequent analyses.

*Histological analysis*: Liver tissue sections were stained with hematoxylin/eosin (H&E) and examined under an optical microscope (microscope: Nikon Eclipse CI; imaging system: Nikon Digital Sight DS-FI2, Tokyo, Japan). The score of liver fibrosis was calculated based on the Metavir scoring system (Bedossa & Poynard, 1996; Liu et al., 2013): grade S0: represented normal liver tissue without fibrosis; grade S1: represented a small number of collagen fibers extending in the portal area or around the central vein, with a relatively intact liver lobule structure; grade S2: represented collagen fibers extending outwards, without formation of pseudolobules; grade S3: represented formed pseudolobules, liver lobule structure disorder, surrounded by fibrous wrapping; grade S4: represented damaged liver structure, diffuse fibrosis, and pseudoglomerulus filled with large and proliferated fibrous tissue. Liver areas with collagen fibrosis were identified by Masson's trichrome staining. The percentage of blue collagen fibers was determined via the Image Pro-plus 6.0 Software and calculated with formula (5) shown below:

(5) $$\text{The collagen fibers area }(\%)=\frac{\text{blue collagen fibers area}}{\text{whole tissue area}}\times 100\%\;$$

*Biochemical analysis*: The levels of AST, ALB, and ALT in serum samples were assessed by a serum biochemical analyzer (BA-88A, Mindray, Shenzhen, China). The levels of SOD, MDA, and Hyp in liver tissues and serum were quantified following instructions on the kit.

### 2.10. Statistical analysis

Data are presented as the mean ± standard deviation. Groups were compared by the paired-sample *t*-test and two-way ANOVA. All statistical analyses were performed using GraphPad Prism 8 (La Jolla, CA). Differences in *p* value *<.05, **<.01, and ***<.001 were considered statistically significant.

## 3. Results and discussion

### 3.1. Synthesis of UA-PEG-SA

Figure 1(B) shows the spectra of the structures of intermediate and final products verified by ^1^H NMR. The signals at *δ* 4.54–4.64 ppm in Figure 1(B) (a) were attributed to the hydrogen attached to the acetoxy group of UA. No signal peaks were observed between *δ* 3.87 and 4.45 (the two hydroxyl groups of UA) (Zhang et al., 2019), demonstrating the acetylation of the carboxyl group on UA. The carboxyl active hydrogen of acetyl ursodeoxycholic acid (Figure 1(B) (a)) was *δ* 11.96 ppm; however, the peak disappeared (Figure 1(B) (b)), indicating complete esterification of the carboxyl group on UA. Additionally, two strong hydrogen signal peaks occurred at *δ* 3.51 and 1.23 ppm (hydrogen of polyethylene glycol and stearic acid, respectively (Figure 1(B) (b)); however, no other active hydrogen signals were reported, which demonstrate successful esterification of the target product UA-PEG-SA.

### 3.2. Optimization of up-AA-NLC and response surface analysis

A three-factor, three-level BBD was employed to optimize UP-AA-NLC. The independent variables and observed responses for each of the 17 runs are listed in Table 2. First-order, second-order, and quadratic models were fitted simultaneously using the Design-Expert software. The comparative values, including *R*, Std. Dev. (SD), *F*-value, and *p* values are shown in Table 3. The statistical data showed that the best-fitting model was quadratic, with the lowest SD value and the highest *R* value. The quadratic equations and 3D response surface plots were generated for each response. Of note, a positive sign in front of a factor in a regression equation indicates that the response is directly proportional to the factor (Narendar & Karthik, 2017). Accordingly, all the three independent variables had interactive effects on zeta potential, particle size, EE, and DL.

**Table 3.** Statistical ANOVA results of the models for the four responses.

Response	Models	Std.Dev.	*R*	*F* Value	*p* Value Prob>*F*
*Y*_1_ (particle size)	Linear	8.42	0.7990	7.65	.0034
	2FI	8.31	0.8539	4.49	.0184
	Quadratic	4.74	0.9687	11.84	.0018
*Y*_2_ (zeta potential)	Linear	2.96	0.6190	2.69	.0894
	2FI	2.93	0.7316	1.92	.1728
	Quadratic	2.10	0.9127	3.88	.0438
*Y*_3_ (encapsulation efficiency)	Linear	4.09	0.7293	4.92	.0168
	2FI	4.19	0.7884	2.74	.0769
	Quadratic	3.40	0.9087	3.68	.0497
*Y*_4_ (drug loadings)	Linear	0.98	0.9780	95.24	<.0001
	2FI	1.09	0.9788	38.01	<.0001
	Quadratic	0.47	0.9973	145.14	<.0001

**Figure 2.**
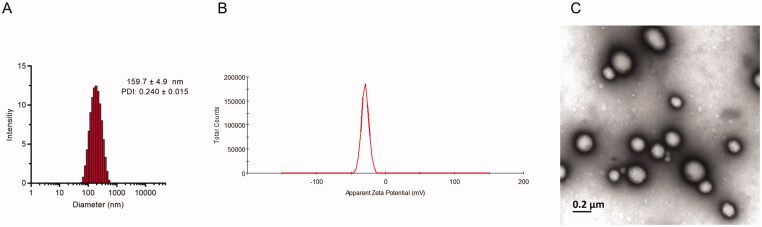
Characterization of UP-AA-NLC. The size (A) and zeta potential (B) distribution of UP-AA-NLC. (C) TEM photograph of UP-AA-NLC.

According to the factorial design, the mean particle size of NPs fluctuated slightly from 150.9 ± 10.7 nm to 195.0 ± 2.9 nm. 3D response surface plots were created as in Figure 2(A). The PEG/lipid ratio was the primary determining factor of the particle size, and it increased in proportion to the particle size. This could be explained by the change in the spatial structure of the NLC after PEG modification, including the formation of a hydrophilic barrier (Wang et al., 2015). The regression equation for particle size and factors is indicated as:

$$Y_{1}=183.20\;+\;3.28A\;+\;13.80B\;+\;1.50C\;+\;5.80AB-4.60AC\;+\;1.75BC-4.90A^{2}-6.15B^{2}-7.20C^{2}$$

Response surface plots in Figure 2(B) demonstrate how independent variables influence the zeta potential of NPs. The absolute value of zeta potential is inversely proportional to the PEG ratio. To explain this relationship, the PEG chains alter the surface hydrophilicity and reduce the surface charge; the PEGylated NLC alters surface properties, including zeta potential (Liu et al., 2015). Moreover, as the OA ratio increases, the absolute value of the potential increases slightly and then decreases. It is suggested that the carboxyl groups of OA and the accumulation of excessive carboxyl groups may reduce negative potential. The regression equation is as follows:

$$Y_{2}=-24.90\;+\;1.75A\;+\;1.19B-2.09C-2.13AB-0.63AC\;+\;1.45BC-2.05A^{2}-0.025B^{2}+\;3.07C^{2}$$

As demonstrated in Figure 2(C,D), EE is negatively correlated with AA, implying that an increase in AA dosage drastically decreases EE. On the contrary, DL and AA are positively correlated. The change in the ratio of PEG and OA slightly impacts EE and DL. The regression equations are as indicated:

$$\begin{array}{c} Y_{3}=83.63-5.30A\;+\;0.96B-1.33C-0.073AB\;+\;3.01AC\\ +\;1.14BC-4.05A^{2}-2.13B^{2}+\;1.12C^{2}\\ Y_{4}=10.94\;+\;5.81A-0.47B-0.059C-0.22AB-0.11AC\\ +\;0.21BC-1.55A^{2}-0.100B^{2}+\;0.31C^{2} \end{array}$$

### 3.3. Formulation optimization for the up-AA-NLC and model validation

Based on the BBD, the optimum percentages were 17% of AA/lipid, 5% of UA-PEG-SA/lipid, and 28% of OA/lipid. Table 4 shows the predicted and experimental zeta potential, particle size, EE, and DL. The absolute value of the deviation between the predicted and experimental values was less than 5%, demonstrating strong predictability of the established model and high reproducibility of the selected formulation.

**Table 4.** Comparison of predicted and experimental values.

Response	Predicted value	Experimental value^a^	Deviation (%)
*Y*_1_	158.1	159.7 ± 4.9	–1.0
*Y*_2_	–27.0	–27.7 ± 1.7	–2.6
*Y*_3_	79.35	77.44 ± 0.69	2.4
*Y*_4_	11.05	10.53 ± 0.10	4.7

^a^ Mean ± SD, *n* = 3.

### 3.4. Characterization of the UP-AA-NLC

**Figure 3.**
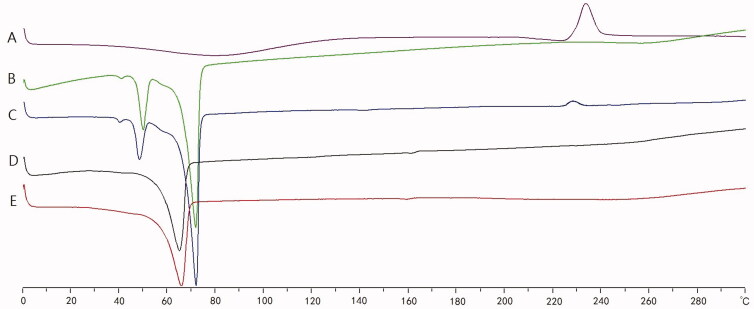
DSC image of (A) AA powder, (B) Ingredients mixtures, (C) Physical mixtures, (D) UP-AA-NLC, (E) P-AA-NLC.

The average particle size of the optimized UP-AA-NLC was 159.7 ± 4.9 nm and a PI of 0.240 ± 0.015, indicating a relatively narrow particle size distribution and uniform nanoparticle size (Figure 3(A)). Zeta potential represents the degree of repulsion between adjacent and similarly charged particles in a dispersion. A greater absolute value, implies a stronger ability to resist particle agglomeration and greater stability of the dispersion. In the present study, UP-AA-NLC exhibited a negative surface charge (–27.7 ± 1.7 mV), demonstrating high stability. The percentage values of EE and DL in UP-AA-NLC were 77.44 ± 0.69% and 10.53 ± 0.10%, respectively (Table 4). The TEM images of the optimized UP-AA-NLC showed that the nanoparticles were mainly spherical (Figure 3(C)) with similar sizes to those detected by Zetasizer.

**Figure 4.**
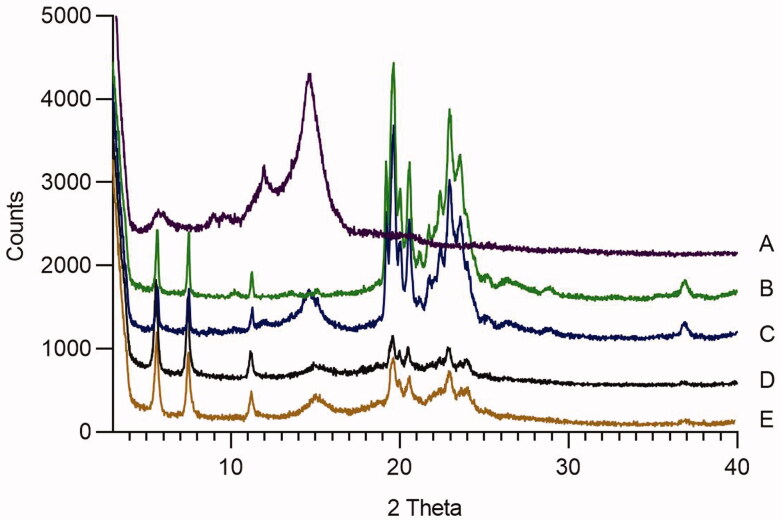
X-ray Diffraction Pattern of (A) AA powder, (B) Ingredients mixtures, (C) Physical mixtures, (D) UP-AA-NLC, (E) P-AA-NLC.

The DSC thermograms of AA powder (A), ingredient mixtures (B), physical mixtures (C), UP-AA-NLC (D), and P-AA-NLC (E) are shown in Figure 4. Of note, AA exhibited a sharp endothermic peak at 233.6 °C. The ingredient mixtures had two melting peaks at 50.2 °C and 71.8 °C. All the peaks were observed in the physical mixtures but with a slight shift. However, the AA peak disappeared from the thermograms of the NLCs (UP-AA-NLC and P-AA-NLC), indicating the conversion of the drug from a crystalline form to an amorphous state.

**Figure 5.**
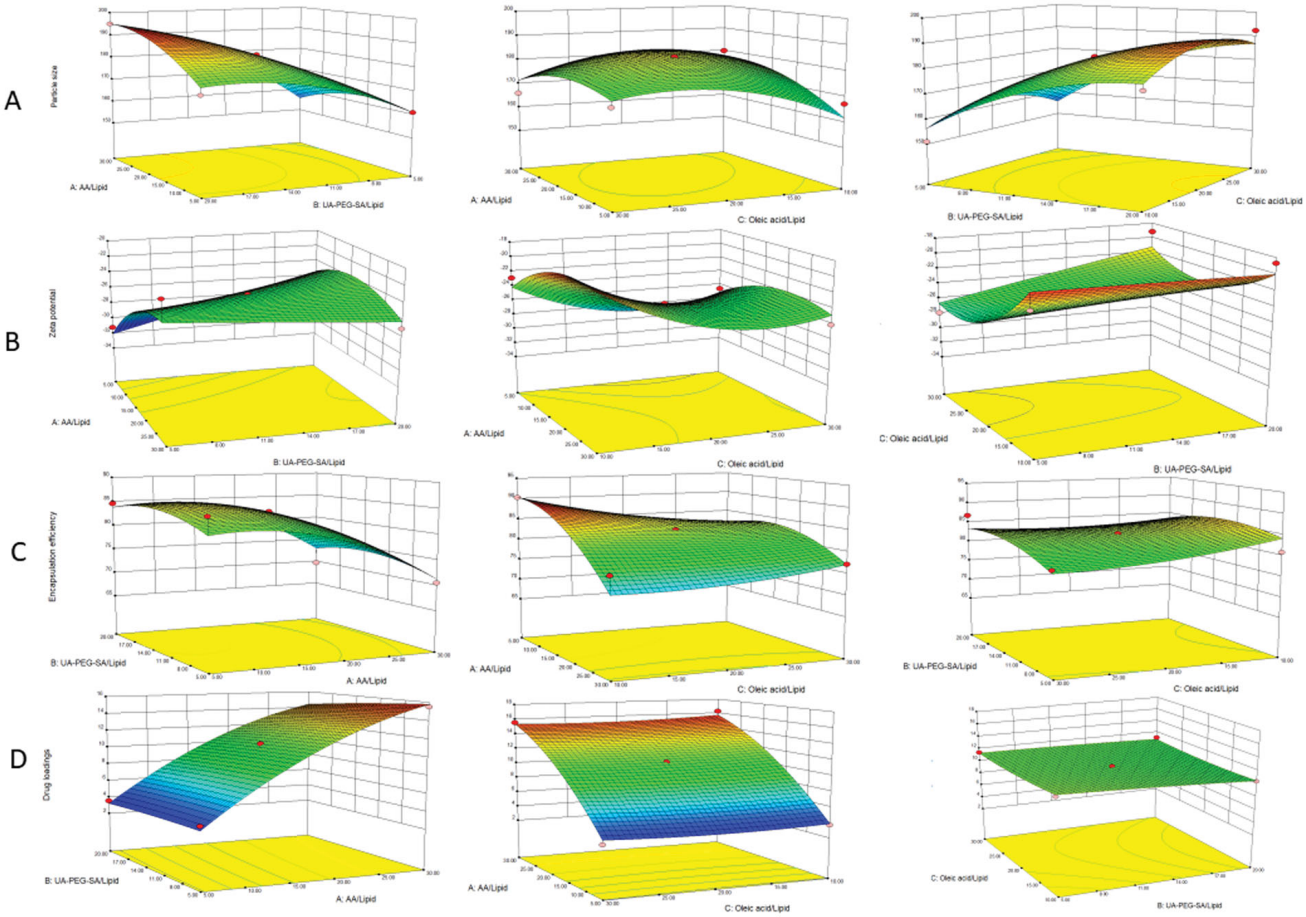
3D Response surface plots showing the effect of independent variables on response parameters. (A) Particle size (Y1), (B) Zeta potential (Y2), (C) Encapsulation efficiency (Y3), (D) Drug loadings (Y4).

To evaluate the microstructure changes of AA in NLCs, XRD was used and the spectra of AA powder (A), ingredients mixtures (B), physical mixtures (C), UP-AA-NLC (D), and P-AA-NLC (E) are displayed in Figure 5. The XRD spectrum of AA powder exhibited sharp peaks at 2*θ* scattering angles from 10° to 16°, indicating AA was crystallized. Both ingredient and physical mixtures had intense diffraction peaks at 2*θ* from 19° to 25°, demonstrating that the lipids were in a stable crystalline state. However, the XRD spectrum of UP-AA-NLC and P-AA-NLC exhibited weak and broad diffusion peaks, which indicated partial loss of crystallinity and suggested that AA was in an amorphous state in NLCs.

**Figure 6.**
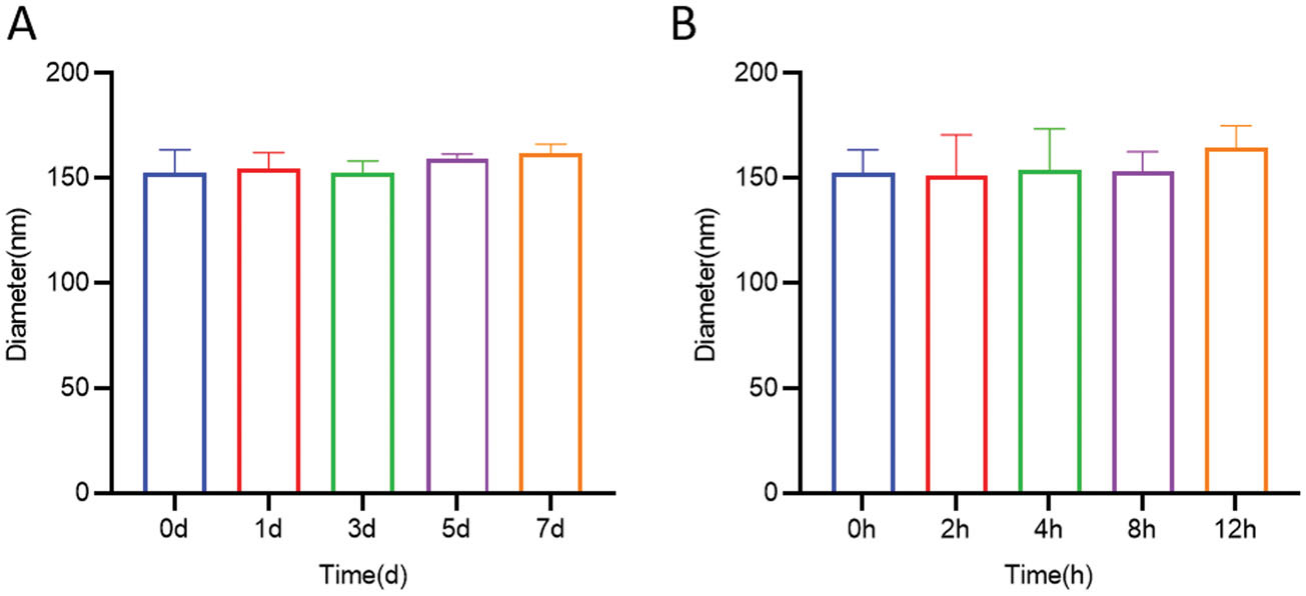
Stability of UP-AA-NLC at 4? and 37? (pH7.4, containing 10% fetal bovine serum) (mean ± SD, *n* = 3).

The average particle size of UP-AA-NLC remained stable at 4 °C for over 12 days (Figure 6(A)). Further evaluation of the stability of UP-AA-NLC was conducted under simulated physiological circulation. Results showed that the change in the size of UP-AA-NLC at 37 °C was not significant, indicating that NLC remained stable in blood *in vivo* within 12 h.

### 3.5. Drug release *in vitro*

**Figure 7.**
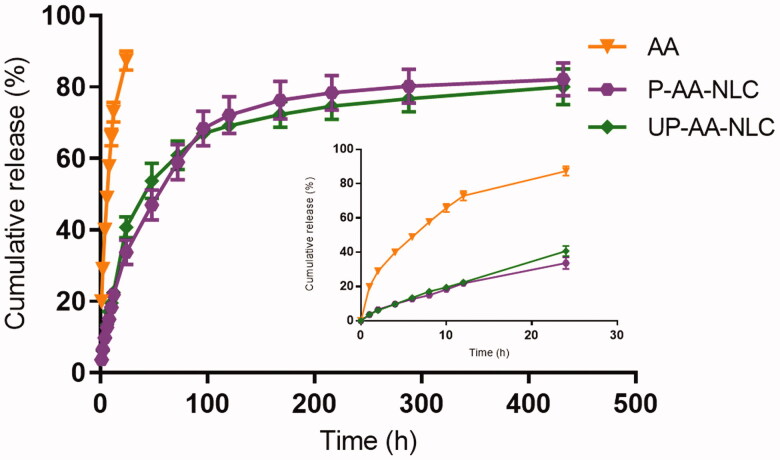
In vitro release profiles of AA, P-AA-NLC and UP-AA-NLC in PBS (pH7.4, containing 1% SDS) (mean ± SD, *n* = 5).

The release patterns of AA, P-AA-NLC, and UP-AA-NLC are illustrated in Figure 7. The AA solution released about 90% within 24 h, while UP-AA-NLC was slower and released 41%, indicating that drug release from the lipid matrix was blocked. The discrepancy in release patterns between AA and NLCs may be attributed to the entrapment of drug molecules in the insoluble lipid matrix. It is noteworthy that the solid shell in the NLCs could protect and immobilize AA in the core of the NLC. Similar drug release behavior was reported for P-AA-NLC and UP-AA-NLC, but neither showed explosive release in the initial stage. This phenomenon demonstrates that the unencapsulated drug was not attached to the surface of the particle.

To clarify the mechanism of AA release from NLCs, the release index n was calculated according to Equation (4). The *n* values of P-AA-NLC and UP-AA-NLC were 0.53 (*R* = 0.982) and 0.52 (*R* = 0.972), respectively, demonstrating that the release mechanism of P-AA-NLC and UP-AA-NLC both followed an abnormal transmission mechanism. These results indicate the possible involvement of diffusion and lipid erosion in controlling the drug release rate of the lipid core and surfactant interface membrane (Wu et al., 2021).

### 3.6. Effects of AA or up-AA-NLC on LX-2 cell proliferation and activation

**Figure 8.**
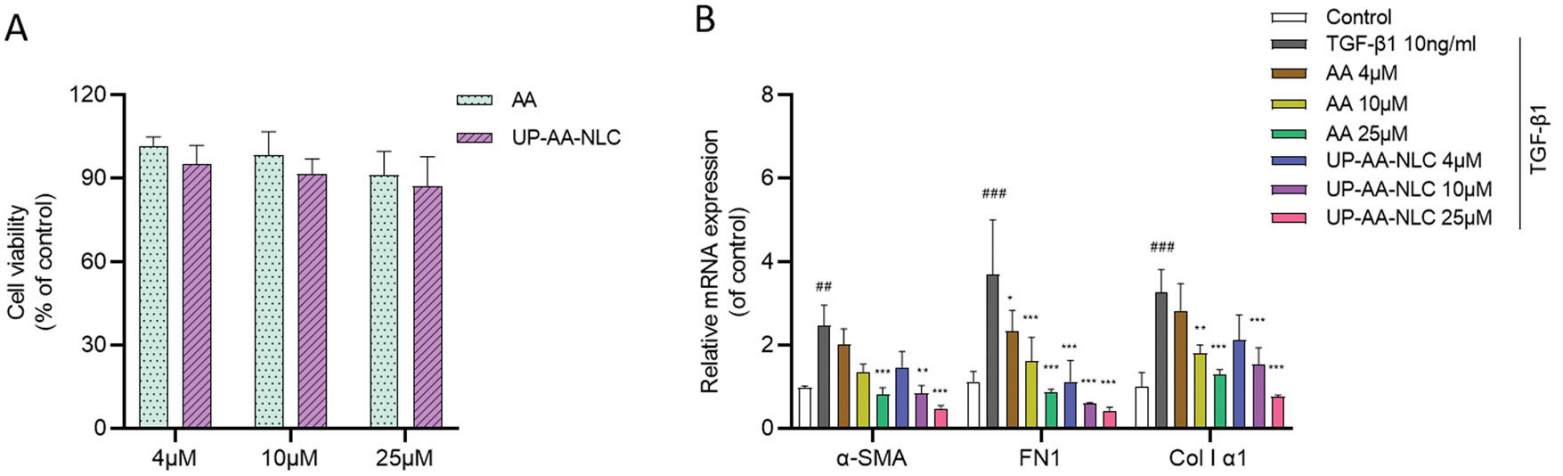
(A) Cytotoxicity of AA and UP-AA-NLC in LX-2 cells. (B) Quantitative real-time PCR analysis of ?-SMA, FN1 and Col I ?1 expression in LX-2 cells. Each column represents the mean ± SD from three independent experiments. ##p < 0.01 vs. Control group, ###*p* < 0.001 vs. Control group, *p < 0.05 vs. TGF-β1 group, **p < 0.01 vs. TGF- β1 group, ***p < 0.001 vs. TGF- β1 group.

A concentration-dependent phenomenon was observed in the cell viability of AA, greater than 91% in concentrations ranging between 4 and 25 μM (Figure 8(A)). However, the cell proliferation rate of the UP-AA-NLC group decreased to 87.24% following an increase in concentration to 25 μM. These data indicate an inhibitory effect of UP-AA-NLC against HSCs and the suppression of the proliferation of LX-2 cells by high-dose NLCs.

To ascertain whether AA and NLCs inhibited the activation of HSCs, LX-2 cells were treated with AA or UP-AA-NLC. Next, the expression of α-SMA, FN1, and Col I α1 was measured by q-PCR. The analysis demonstrated that AA and NLCs exert potential antifibrotic effects by decreasing HSC proliferation and inhibiting HSC myofibroblast differentiation. These results were confirmed by the decreased expression of α-SMA, FN1, and Col I α1 (Figure 8(B)). Compared to the AA group, incubation with UP-AA-NLC significantly downregulated the mRNA levels of α-SMA, FN1, and CoL1A1 in LX-2 cells treated with TGF-β1 (Figure 8(B)). The findings suggest that UP-AA-NLC further attenuated TGF-β1-induced proliferation and activation.

### 3.7. Biodistribution and *in vivo* imaging

**Figure 9.**
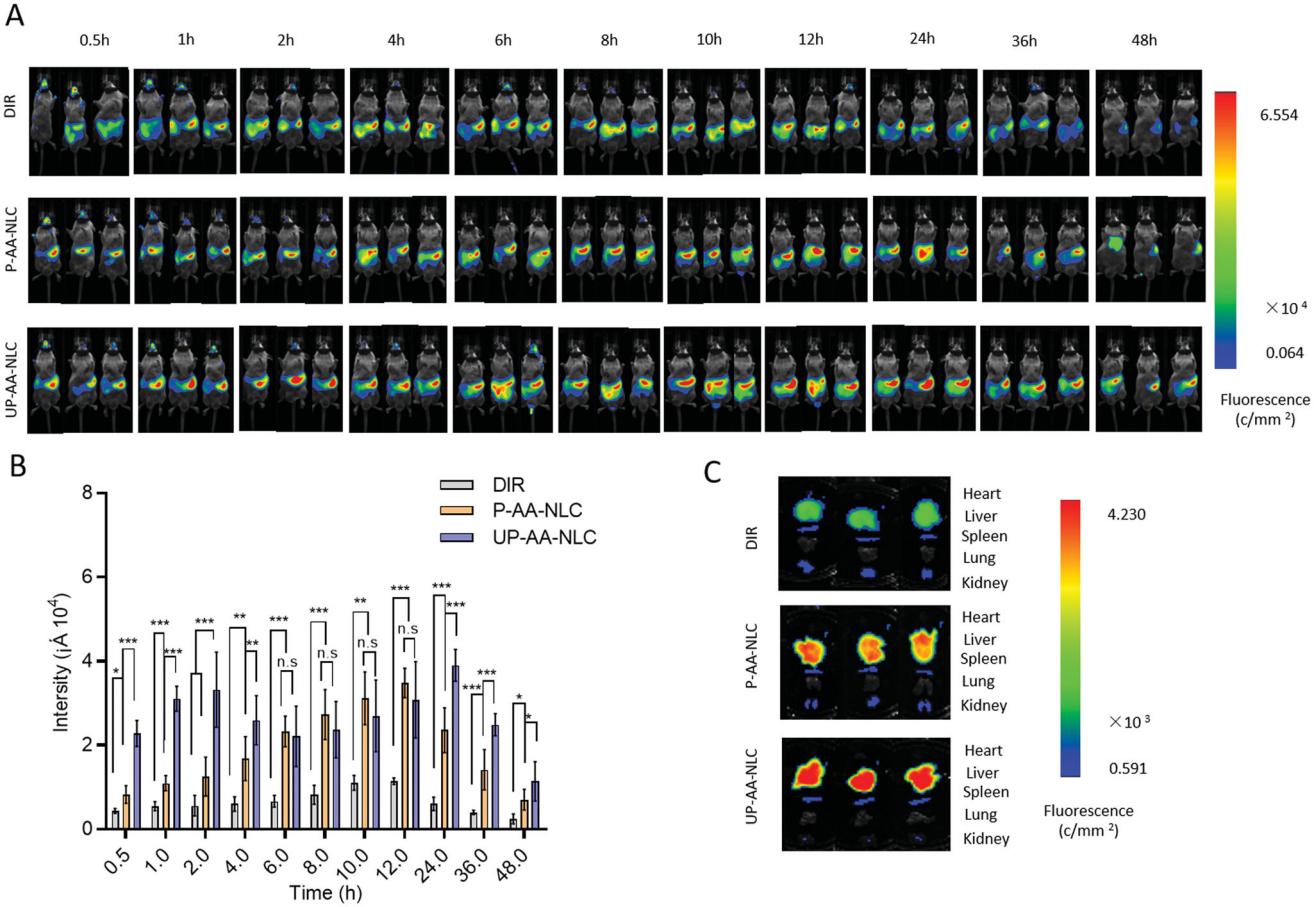
In vivo imaging of AA, P-AA-NLC and UP-AA-NLC biodistribution in rats. (A) Whole body imaging from 0 to 48 h. (B) The fluorescence intensity of the liver sites. (C) The imaging of the dissected major organs. *p? 0.05, **p? 0.01, ***p? 0.001.

Figure 9(A) shows the real-time distribution of DIR fluorescence in living mice after oral administration of DIR solution, DIR-loaded AA-NLC, and DIR-loaded UP-AA-NLC. Results showed DIR accumulation in the liver, and weakened fluorescence intensity after 24 h, indicating that DIR was metabolized. The visible fluorescence accumulation of the AA-NLC group increased gradually in the liver after 30 min and peaked at 12 h (Figure 9(B)). Results demonstrated that nanoparticles potentially prolong drug retention duration *in vivo* and improve oral absorption. The concentration of DIR fluorescence in the liver increased rapidly after modification with UA, in which the first absorption peak was observed at 2 h. Subsequently, the concentration decreased slowly and reached the second peak at 24 h; however, the fluorescence value was still detectable at 48 h. The two peaks of UP-AA-NLC may be related to the involvement of UA in enterohepatic circulation, which successively increases the circulation time of the drug in the body. Ursodeoxycholic acid is a type of bile salt transporter ligand that binds to the ASBT receptor of intestinal epithelial cells. Research shows that AA can cause a significant increase in drug concentrations in the body after oral administration (Sievanen, 2007). Pictures shown in Figure 8(B) are based on a semi-quantitative analysis of the ROI in the liver performed using the built-in software, which also confirms the preceding conclusions. The 48-h *ex vivo* image of resected organs (Figure 9(C)) provided evidence that the DIR-loaded UP-AA-NLC group had a greater fluorescence signal in the liver than the DIR solution and DIR-loaded AA-NLC groups. This could be because the specific binding of UA to the NTCP receptor of hepatocytes allows for selective accumulation of the drug in the liver and long-term retention (Sievanen, 2007).

### 3.8. Tissue distribution study

There was no evidence of endogenous interference in detecting of AA (10.8 min) or IS (19.4 min) in the heart, liver, spleen, lung, or kidney homogenates. Acceptable linear relationships were found in all the tissues over the AA concentration ranging from 0.1 to 8 μg/mL. The calibration curves were as indicated: *y* = 0.1258*x* – 0.0135 (*r* = 0.9992, heart), *y* = 0.1204*x* – 0.0137 (*r* = 0.9991, liver), *y* = 0.1332*x* – 0.017 (*r* = 0.9996, spleen), *y* = 0.1238*x* – 0.008 (*r* = 0.9997, lung), and *y* = 0.1146*x* – 0.005 (*r* = 0.9993, kidney). The lowest concentration on the standard curve (denoted as LLOQ) was 0.1 μg/mL, and the RSD of all tissue homogenates was less than 8%. These results demonstrated the method was sufficiently sensitive to meet the *in vivo* biological analysis requirements of AA. The intra- and inter-day precision and accuracy of QC samples at 0.5, 2, and 4 μg/mL are summarized in Table 5. The recoveries of the three levels in all tissue homogenates were above 70%, indicating that the extraction method was acceptable. The stability of AA in tissues was tested on 5, 15, and 30 days at concentrations of 0.5, 2, and 4 μg/mL, respectively, under storage at −20 °C. The samples were analyzed and compared to freshly prepared QC samples. The RSD values of AA concentration in organs were less than 6%, demonstrating that AA was stable in the heart, liver, spleen, lung, and kidney homogenates.

**Table 5.** Precision, accuracy, and recovery for assay of AA in various tissue homogenates (mean ± SD, *n* = 5).

Samples	Conc. (μg/mL)	Extraction recovery (%)	Accuracy (%)	Relative standard deviation (%)	
				Intra-day	Inter-day
Heart	0.5	77.19 ± 7.51	100.92 ± 6.24	5.13	5.51
	2	74.54 ± 6.93	99.95 ± 3.43	4.82	4.74
	4	88.26 ± 6.74	101.52 ± 3.65	3.68	5.46
Liver	0.5	75.62 ± 5.45	98.51 ± 3.44	6.36	5.28
	2	71.17 ± 3.44	101.89 ± 7.06	6.36	7.22
	4	76.34 ± 2.03	97.55 ± 9.71	4.7	7.16
Spleen	0.5	77.26 ± 4.64	95.80 ± 4.03	5.71	8.13
	2	73.08 ± 5.48	101.39 ± 5.15	3.22	4.95
	4	72.24 ± 3.77	99.00 ± 3.17	2.20	2.79
Lung	0.5	74.25 ± 1.89	100.25 ± 4.13	4.30	8.89
	2	74.61 ± 6.97	99.85 ± 6.24	2.72	4.95
	4	76.82 ± 7.70	103.47 ± 2.63	2.43	3.58
Kidney	0.5	74.17 ± 5.22	101.27 ± 4.60	7.44	5.21
	2	73.88 ± 4.01	96.44 ± 4.02	3.82	4.04
	4	73.34 ± 5.29	102.82 ± 2.87	4.59	3.31

**Figure 10.**
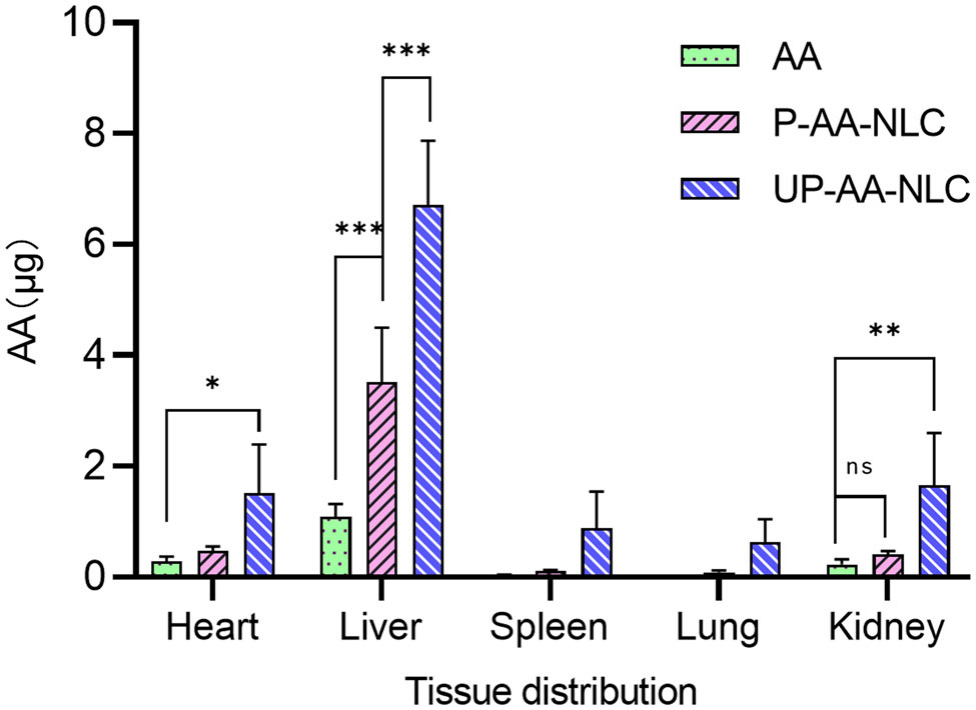
10 Mean concentration of AA in various tissues at 24 h after oral administration at dose of 64 mg/kg (*n* = 3). *p? 0.05, **p? 0.01, ***p? 0.001.

The presently established method was applied to investigate the distribution of AA and NLCs in tissues in rats. Results for drug concentrations in the heart, liver, spleen, lung, and kidney at 24 h following oral administration of AA, P-AA-NLC, and UP-ALC, are shown in Figure 10. Of note, AA was mainly distributed in the liver, heart, and kidney, supported by previously reported distribution characteristics (Yin et al., 2012). Also, NLC potentially improves the absorption of AA. The organ concentration increased with further modification of UA. Among these, the concentration in the liver increased the most, by 6.2 times that of the AA group and 1.9 times that of the P-AA-NLC group; these results largely concurred with the conclusions of *in vivo* imaging.

### 3.9. Anti-liver fibrosis therapy *in vivo*

**Figure 11.**
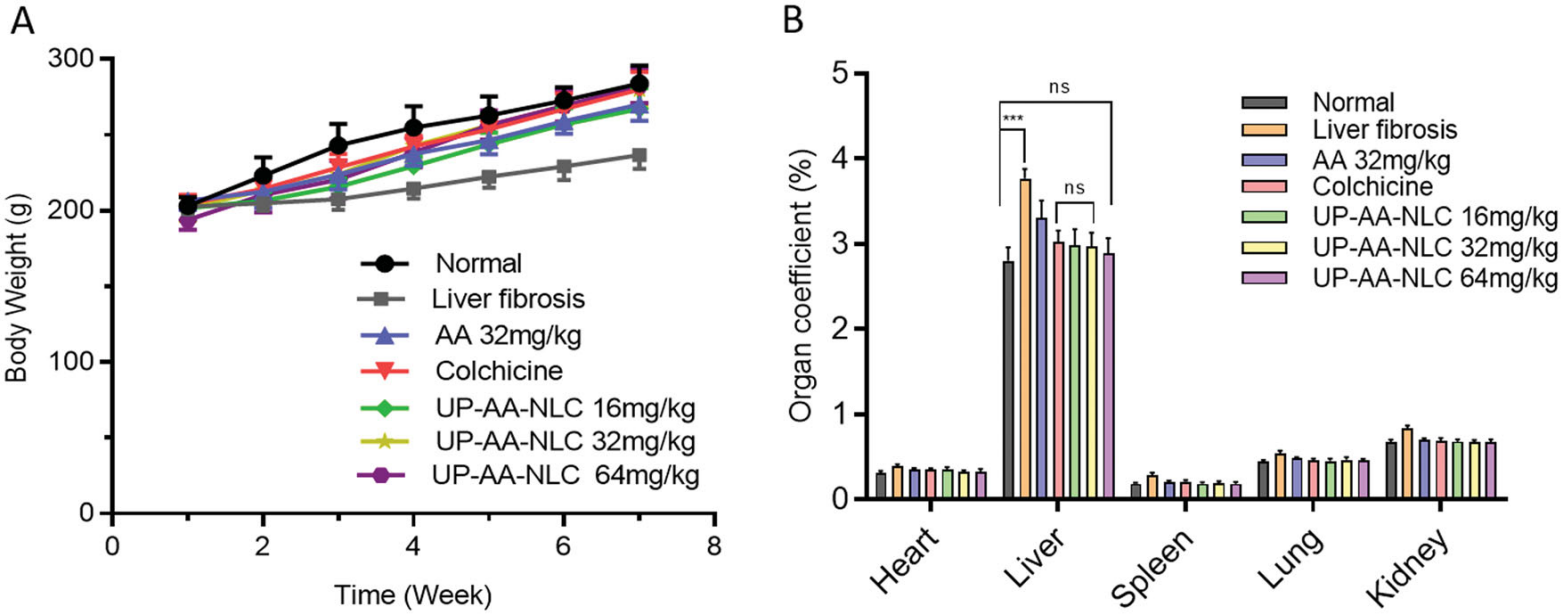
Body weight curve (A) and organ coefficient (B).

Pharmacodynamics studies were performed in the rat model of CCl_4_-induced liver fibrosis. After six weeks of CCl_4_ administration, weight gain in the model group (liver fibrosis group) was slow, but similar to that of normal rats in the other groups (Figure 11(A)). The organ coefficients of each group are shown in Figure 10(B). Compared to the normal group, the liver coefficient of the model group was elevated drastically (*p*<.001), but later decreased post AA administration. The results suggest that AA has a protective effect on liver fibrosis, which is consistent with the conclusion of references (Gao et al., 2006; Tang et al., 2012). The protective effect may be related to the regulation of PI3K/AKT/mTOR and Bcl-2/Bax signaling pathways (Wei et al., 2018). The liver coefficient in colchicine and UP-AA-NLC groups was significantly reduced; however, the difference to the normal group was not significant (*p*> .05).

**Figure 12.**
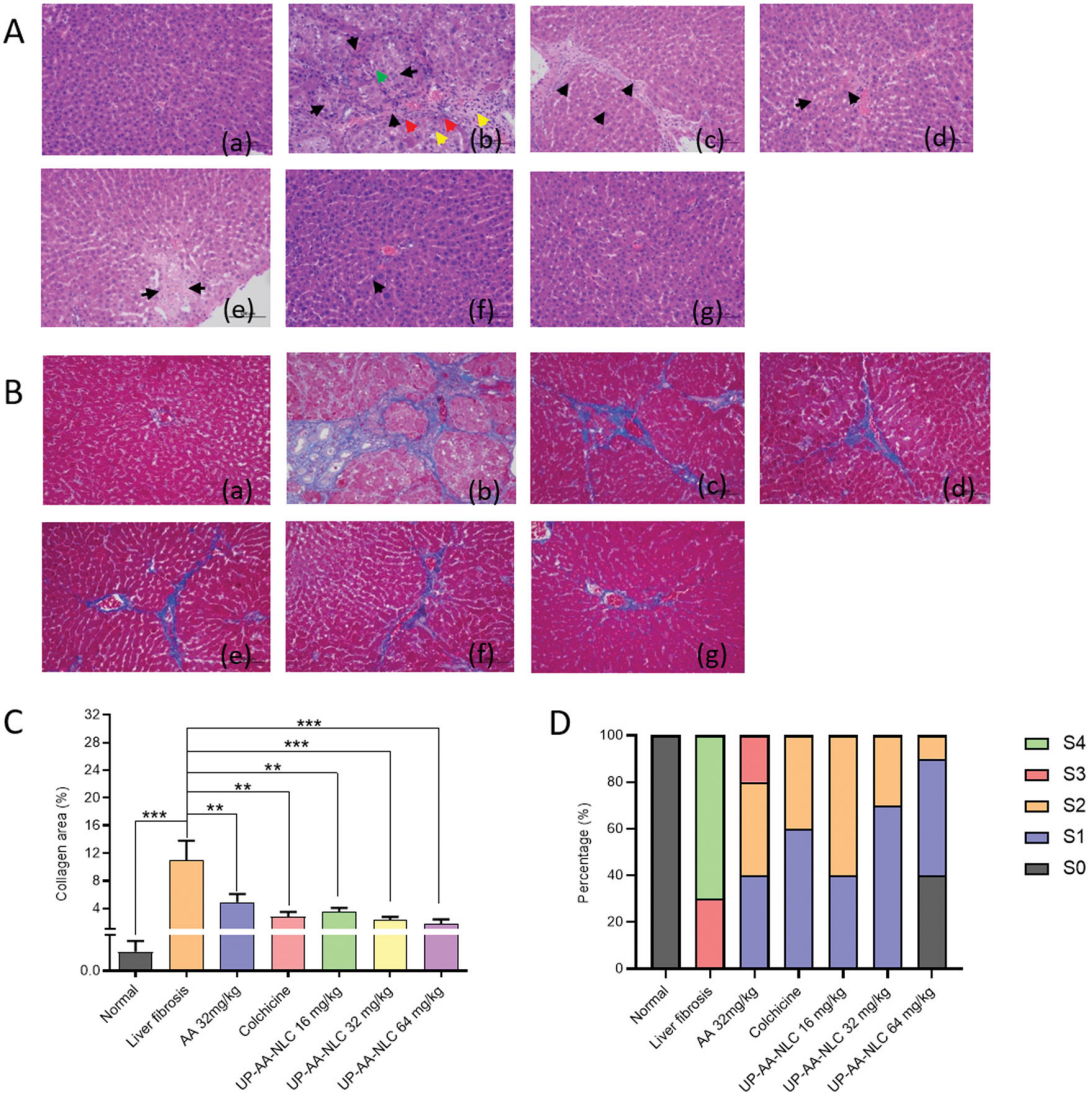
Histopathological analysis of anti-fibrosis efficacy. The hematoxylin-eosin (H&E) staining (A) and Masson staining (B) examination of the dissected livers at the treatment endpoint (scale bar: 200 μm). (a) Normal group; (b) Liver fibrosis group; (c) AA (32 mg/kg); (d) Colchicine; (e) UP-AA-NLC (16 mg/kg); (f) UP-AA-NLC (32 mg/kg); (g) UP-AA-NLC (64 mg/kg). (C) Statistical analysis of liver tissue collagen fiber area after treated with AA, colchicine and UP-AA-NLC. (D) Liver fibrosis grading for all rats according to histopathological results. Grade S0 was defined as healthy liver and from S1 (slight fibrosis) to S4 (severe fibrosis) was represented the severity of liver fibrosis.

The H&E staining examination revealed pathological morphological changes in the liver of rats after injection of CCl_4_. Compared to the normal liver (Figure 12(A) (a)), liver necrosis in the model group was more severe (Figure 12(A) (b)) due to hyperplasia of collagen fibers, formation of pseudolobules (black arrow), and inflammation (red arrow). Moreover, it can also be seen that there were small bile ducts proliferating in collagen (yellow arrows) and cytoplasmic round fat vesicles (green arrows). Colchicine, as the positive control, effectively protected the liver and repaired the CCl_4_-induced damage. Figure 12(A) (d) shows hyperplasia of collagen fibers around the veins with marked extension (black arrow), demonstrating better granules in the liver tissue. Furthermore, the improvement of liver steatosis and fibrotic diaphragm (black arrow) showed the anti-liver fibrosis effect of AA (Figure 12(A) (c)). The liver morphology of the UP-AA-NLC group at low (16 mg/kg), medium (32 mg/kg), and high doses (64 mg/kg) respectively, is shown in Figure 12(A) (e–g). According to the images, the outward extension of collagen fibers became less pronounced as the dose increased. At the dosage of 64 mg/kg, the liver morphology was similar to the normal group. These results suggest that UP-AA-NLC can effectively prevent and reverse CCl_4_-induced chronic liver injury.

Excessive ECM deposition is a crucial feature of liver fibrosis, and collagen is a major component of ECM. In the present study, Masson staining detection of collagen expression in rat liver revealed significant collagen accumulation in liver damaged by CCl_4_. The collagen fiber area in the model group was over 10% (Figure 12(Bb,C)) but reduced to 2.8% in the colchicine group. The UP-AA-NLC group had significantly less liver fibrosis and less accumulation of collagen fibers, which was comparable to or better than the colchicine group. Furthermore, the collagen fiber area of UP-AA-NLC decreased to 32% (*p* < .01), 21% (*p* < .001), and 16% (*p* < .001) of the model group at low, medium, and high concentrations, respectively.

As shown in Figure 12(D), the liver fibrosis scores mainly dominated in grades S4 (70%) and S3 (30%) in the model group and grades S1 (60%) and S2 (40%) in the colchicine treatment group. Compared to the AA group, under the same dose (32 mg/kg), the UP-AA-NLC group had significantly less liver fibrosis, reported in grade S1 (70%). It was reported that P-AA-NLC without UA modification was mainly classified as S2 (50%) at 32 mg/kg (Chen et al., 2020), which also confirmed the protective effect of UA on the liver. Of note, the effect was more pronounced when the dose was increased, and the presence of S0 levels indicated a significant reduction in liver disease. The findings demonstrate that UP-AA-NLC can alleviate CCl_4_-induced liver fibrosis.

**Figure 13.**
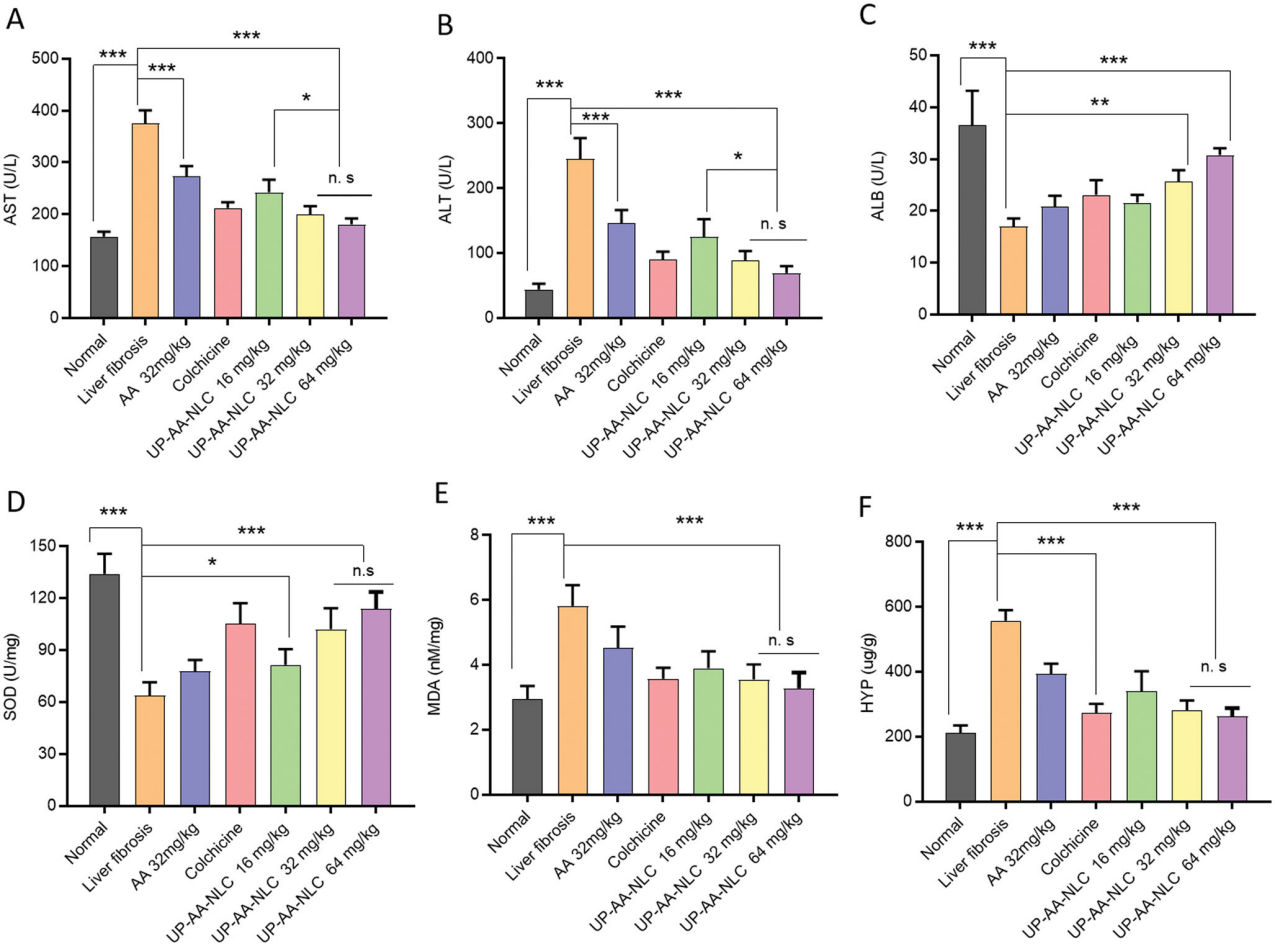
Biochemical analysis of anti-liver fibrosis efficacy. The serum biochemical parameters of AST (A), ALT (B), and ALB (C), and the liver tissue biochemical parameters of SOD (D), MDA (E), and HYP (F).

Serum biochemical marker enzymes, viz. AST and ALT levels were increased significantly, and the ALB level was decreased significantly in the model group compared to the normal group (238.92 ± 16.38%, 558.54 ± 72.75%, and 46.58 ± 4.28% of the normal control, *p* < .001) (Figure 13(A–C)). These findings indicate that CCl_4_ successfully induced liver fibrosis. Treatment with UP-AA-NLC reduced liver functional impairment in a dose-dependent manner, with significant effects at doses of 32 and 64 mg/kg. The 64 mg/kg dose group of UP-AA-NLC had better efficacy than the colchicine group. The values of AST, ALT, and ALB were 115.29 ± 7.14%, 157.86 ± 24.37%, and 84.38 ± 3.61% of the normal group, respectively.

The measurement results of anti-oxidant activity parameters are shown in Figure 13(D,E). Compared to the normal group, CCl_4_ treatment effectuated a significant decrease in SOD and a remarkable increase in MDA (*p*<.001). Among these treatments, the UP-AA-NLC 64 mg/kg group exhibited the most significant effect on the elevation of SOD and reduction of MDA. These results provide evidence that UP-AA-NLC can effectively inhibit liver fibrosis by blocking oxidative stress. HYP is a unique amino acid composition of collagen, and its abundance in the liver reflects changes in collagen metabolism (Shkurupii et al., 2014). Herein, treatment with colchicine, AA, and its NLCs drastically lowered HYP levels in the fibrotic liver (Figure 13(F)), indicating that collagen accumulation in the fibrotic liver was decreased. Overall, these data demonstrate that UP-AA-NLCs potentially attenuate CCl_4_-induced liver fibrogenesis by suppressing collagen expression.

## 4. Conclusions

UA-modified UP-AA-NLC has been evaluated as a potential oral drug delivery system that potentially promotes liver targeting abilities and antifibrotic therapeutic efficacy. The formulation of nanoparticles was optimized using the BBD design based on the size, zeta potential, DL%, and EE% results. TEM images revealed that the produced NLC was spherical and uniform in size. The slow-release characteristics of NLC indicated uniform encapsulation of AA in the lipid matrix. *In vitro*, anti-fibrosis activity and proliferation of LX-2 cells showed that UP-AA-NLC significantly promoted TGF-beta1-induced α-SMA, FN1, and Col I α1 expression. *In vivo* biodistribution studies revealed that UP-AA-NLC exhibited liver-targeting capabilities and potentially improved oral absorption of the drug. Also, UP-AA-NLC significantly reduced CCl_4_-induced liver fibrosis and functional damage in a dose-dependent manner after oral administration. Findings from this work suggest that AA nanoparticles, modified from UA, are potential drug delivery carriers in liver-targeted therapy and sustained drug release.
